# AirQo sensor kit: A particulate matter air quality sensing kit custom designed for low-resource settings

**DOI:** 10.1016/j.ohx.2023.e00482

**Published:** 2023-11-02

**Authors:** Engineer Bainomugisha, Joel Ssematimba, Deogratius Okedi, Anold Nsubuga, Marvin Banda, George William Settala, Gideon Lubisia

**Affiliations:** AirQo, Department of Computer Science, Makerere University, Kampala, Uganda

**Keywords:** Air quality sensing, Autonomous environmental sensing, African urban air quality monitoring

## Abstract

Air pollution remains a major public health risk. People living in urban spaces are among those most affected by exposure to unhealthy levels of air pollution. However, many urban spaces especially in low- and middle-income countries lack high resolution and long-term data on the state of air quality. Without high resolution air quality data on the different spaces in a city, citizens and authorities are unable to quantify the challenge and act. This is in part attributed to the high cost of air quality monitoring equipment that are expensive to set up, maintain and not designed for local operating conditions that characterise environments in such contexts. In this paper, we describe AirQo sensor kit, a low-cost sensing hardware system designed for and custom made to work in low-resource settings and outdoor urban environments. We describe the design of the air quality sensing device, 3D-printed enclosure, installation-mount, fabrication and deployment configurations. We demonstrate that the low-cost sensing hardware provides a complete solution comparable to the traditional monitoring system and inspires action to tackle air pollution issues. The sensor kit presented in this paper has been widely deployed in cities in Eastern, Western and Central African countries.

## Graphical abstract

**Figure:**
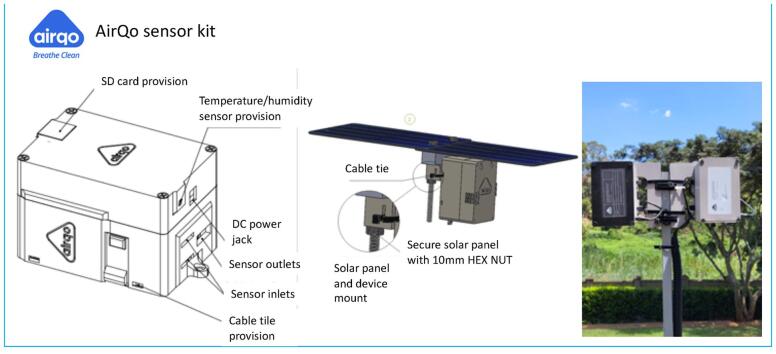

Specifications table:

**Table:** 

Hardware name	*AirQo sensor kit*
Subject area	Environmental sciences; Educational tools and open source alternatives to existing infrastructure
Hardware type	Field measurements and sensors; Electrical engineering and computer science
Open source license	CC BY 4.0
Source file repository	https://doi.org/10.17632/zrjbtfhwh9.2

## Hardware in context

Measurement of air quality is an important dataset for sustainable environmental management of urban spaces. The quality of air that people breathe is an important metric for the quality of life in many spaces. The World Health Organization (WHO) reports show that over seven million people die every year worldwide due to illness linked to poor air quality exposures [1]. Traditional monitoring of air quality is by installing fixed equipment in specific locations of interest. For example, major-roads, schools, industrial areas and residential areas. Despite the availability of these equipment, many urban spaces in low- and middle-income countries (LMICs) remain unmonitored. The limited access and availability of air quality monitoring devices and datasets has also inhibited research into the understanding of air quality and its effects on health in the contexts.

The major barriers and constraints to air quality management include the cost of setting the monitoring infrastructure, the local capacity to set up and maintain the air quality monitoring systems, the fit for the environment and operational contexts [2], [3], [4], [5]. Traditional air quality monitoring equipment, for example, requires access to reliable power supply, which may not be available in the would-be suitable deployment locations. Monitoring networks may also require means to transmit data. This requires reliable Internet infrastructure, which is not always available in locations where air quality monitors are installed. Where power and Internet are available, they may be intermittent requiring specific hardware and communications engineering skills to circumvent the challenges.

Low-cost sensing technologies (LCSTs) are emerging as complementary approaches to the traditional air quality monitoring [6], [7]. LCSTs offer new possibilities for the LMICs because they allow for the setup of high-resolution networks at a lower cost than traditional reference monitors. However, the operating environments and contexts in urban spaces of LMICs present unique hardware and software requirements [5]. There are limited examples of hardware design and sensing systems developed for these contexts. We hypothesise that the capacity in these settings may remain low without in-context initiatives and examples of open hardware frameworks and systems designed for the low- and middle-income settings. In the previous work [5], we presented the design considerations for designing, deploying and managing a large-scale air quality sensor network in low-resource settings. This paper extends that work by providing an in-depth presentation of the design and validation of hardware and firmware for the AirQo sensors to enable replication of the system.

In this paper, we describe the AirQo sensor kit, a low-cost sensing hardware and air quality sensing system that is custom designed to work in environments of cities in low-resource settings. We describe the design of the hardware, 3D-printed enclosure, installation mount and deployment configurations. We demonstrate that the low-cost sensing hardware provides a complete solution to the traditional monitoring system and inspires action to tackle air pollution issues. The AirQo sensors have been evaluated against the reference grade monitors in field deployments and show a strong correlation between the two devices for the measurement of particulate matter (PM) PM_2.5_ and PM_10_.The AirQo devices have been field validated and deployed in several African countries including Uganda, Kenya, Nigeria, Senegal, and Cameroon.

## Hardware description

**Fig. 1:**
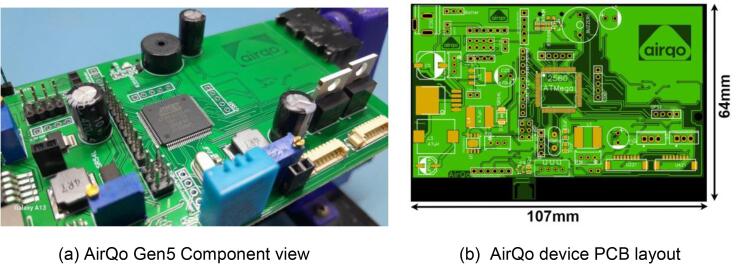
AirQo component view and PCB layout.

The AirQo device (*Gen. 5 Rev. B.2*) is an IoT enabled air quality monitoring device that measures particulate matter PM_2.5_ and PM_10_ concentrations with dual PMS5003 sensors using laser scattering technique for the measurement of particle size and concentration. The purpose for the dual particulate matter sensors is to enhance checks and controls on the data reliability and quality. The device uses SIM800L Global System for Mobile Communications (GSM) (which is readily available in places of deployment in many settings) to relay data wirelessly to a cloud-based datastore [5]. The collected data is calibrated using cloud-based machine learning methods [8]. AirQo monitors are designed to be low-cost, lower power consumption devices. The AirQo monitor’s compact volume allows it to blend in the environment and collect reliable air quality data with minimum interaction. The AirQo monitor is built from quality, low cost and readily available materials. Fig. 1 shows the AirQo component overview and the printed circuit board (PCB) layout.

The AirQo monitor runs on an atmega2560 microcontroller that is paired with a dual plantower PMS5003 [25]. The device includes UBLOX NEO-6 M GPS to localise the data collected, DHT11 to offer data insights into the internal device conditions and an SHT25 for external temperature and humidity reading. The DHT's elevated and off-board orientation enables precise and accurate readings on the internal device performance. Also, the SHT25 is less subject to noise with higher accuracy for precise and accurate recording of external temperature and humidity readings. The device supports a wide input voltage, 5 V-12 V using a high frequency switching regulator at the power input and can be powered using both mains power and solar power. Acceptable current is up to 5 Amps. The monitor has a battery that allows it to extend its operations in the night in the case when powered with solar or during prolonged power cut-outs. The device is powered by a 4000mAH Li-ion battery back, charged by two 6 W solar panels connected in parallel. The enclosure is specially designed to minimise interferences from the dust, insects, and rain.

**Fig. 2:**
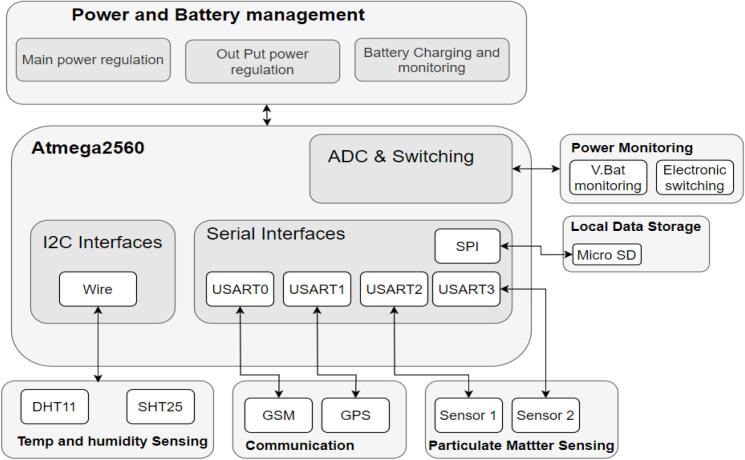
AirQo device hardware overview.

Fig. 2 shows the overall overview of the AirQo devices and sensing components. The AirQo device is also designed to be portable to allow for installation on static objects such as electric poles or mobile objects such as motorcycle taxis. It is significantly lower cost compared to reference grade monitors that cost thousands of USD making them inaccessible for cities in low- and middle-income countries. Setting up a traditional air quality monitoring costs in the range of USD 50,000 and USD 250,000 [9]. Traditional monitoring stations also occupy a large volume space and require lots of energy/power to function well. Data access methods for these monitors come at high cost, normally paying thousands in dollars for data collection tools, and often requiring manual collection.

The AirQo sensor kit has many advantages over existing air quality sensing systems particularly when considered for deployment in the context of low-resource settings and tropical weather conditions [5]. The AirQo sensor kit includes an integrated calibration and quality assurance model as part of the data pipeline resulting in a quality assured and calibrated dataset. Many of the existing sensor construction experiments are short lived and have not deployed and validated in the field at a large scale. AirQo system has been validated and refined 2015–2023 [4], [5], [8], [19]. The AirQo casing is uniquely designed to maximise air flow optimised to maximise airflow. It includes venting larger than the PM sensor vent to reduce resistance in airflow. The AirQo sensor kit is designed to minimise the deposition of dust and insect infestation. While dust may not be an issue in other settings, we found it to be a major issue for deployments in African cities. Due to the higher number of unpaved roads in low-resource settings, sensor kits that are not designed to cater for this design requirement are negatively impacted on by clogging due to high levels of dust. The design of the AirQo sensor kit is provided in the design files in Table 1. The AirQo sensor kit is small in size and portable allowing for flexible deployment options for example for mobile and fixed monitoring. The AirQo devices have flexible power options including solar, mains, motorcycle battery and alternator. To the best of our knowledge, there are no previous air quality sensors that have been field-validated and calibrated in a spatial and longitudinal scale as large as the AirQo system in African environments.

**Table 1:** Summary of the design files for the AirQo sensor kit.

**Design file name**	**File type**	**Open source licence**	**Location of the file**
Main body enclosure (this houses the battery and PM sensors)	STL	CC BY 4.0	https://doi.org/10.17632/zrjbtfhwh9.2
Main Cover enclosure (this holds the PCB)	STL	CC BY 4.0	https://doi.org/10.17632/zrjbtfhwh9.2
Power jack Cover	STL	CC BY 4.0	https://doi.org/10.17632/zrjbtfhwh9.2
PM Sensor Holder (this enables easy installation and removal of the PM sensors)	STL	CC BY 4.0	https://doi.org/10.17632/zrjbtfhwh9.2
SD Cover (a cover to protect the SD-card)	STL	CC BY 4.0	https://doi.org/10.17632/zrjbtfhwh9.2
Hardware Schematics	PDF	CC BY 4.0	https://doi.org/10.17632/zrjbtfhwh9.2
PCB View Top	SVG	CC BY 4.0	https://doi.org/10.17632/zrjbtfhwh9.2
PCB View Back	SVG	CC BY 4.0	https://doi.org/10.17632/zrjbtfhwh9.2

## Design files

Table 1 presents the various design files required for the construction of the AirQo sensor kit. The design files names in the table correspond to the file names in the repository.

**Fig. 3:**
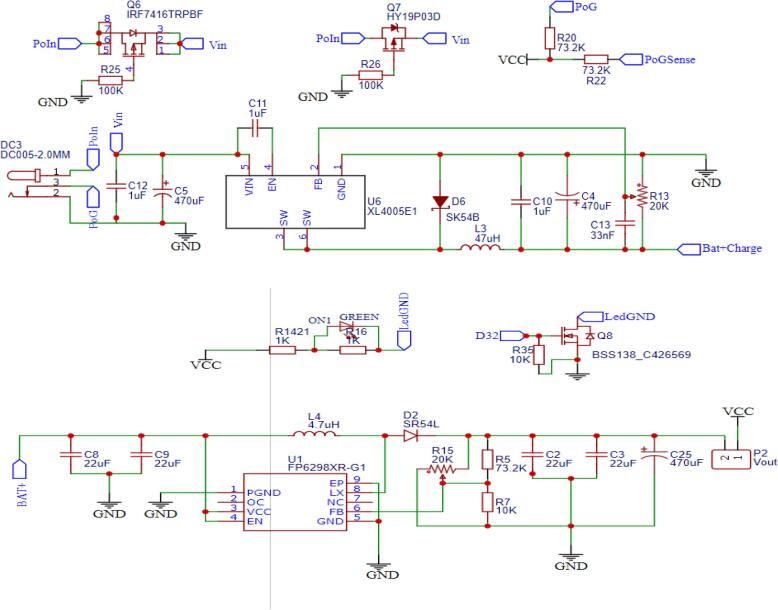
Power Schematics.

The design files consist of all the needed content to manufacture, assemble the AirQo Gen5 device, the repo will allow access to schematics designs which are grouped into power elements, sensing elements, local storage, communications and item switching. The device is designed to manage any DC supply ranging between 5.0 V and 15.0 V DC. The power circuitry of the device includes a high efficiency switch mode step down that converts the DC to a suitable low voltage (Fig. 3). The power circuitry also provides reverse charge protection by use of a series of mosfets (P-Channel). Noise filtration is done before and after stepping down the DC voltage using a series capacitor. A range of Lithium batteries can be attached and charged using a suitable charge voltage, the chips selected provide a good Constant Current Constant Voltage (CCCV) that is good for use with Lithium ion batteries.

**Fig. 4:**
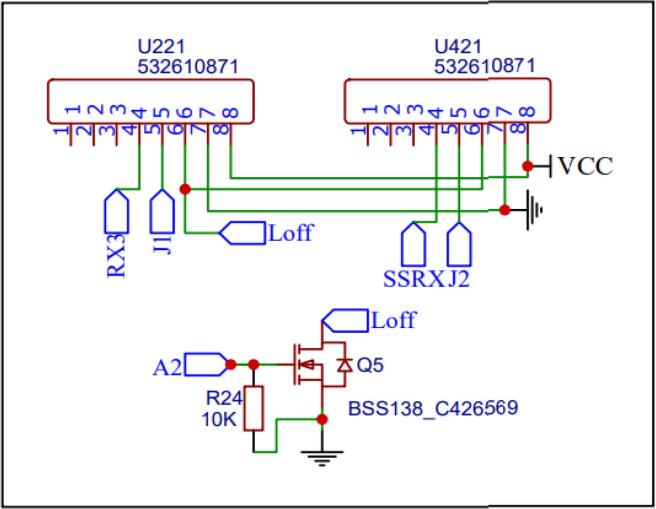
PM Sensor Schematic connection.

The device uses two PMS5003 sensors for particulate matter counting. These use a USART connection to communicate with the microcontroller at a set baud rate (9600). Connectors (U221, U421) provide an interface to connect the PMS5003 sensors using dedicated cables. Transistor Q5 enables switching of the sensors by the microcontroller through a digital pin (Fig. 4).

**Fig. 5:**
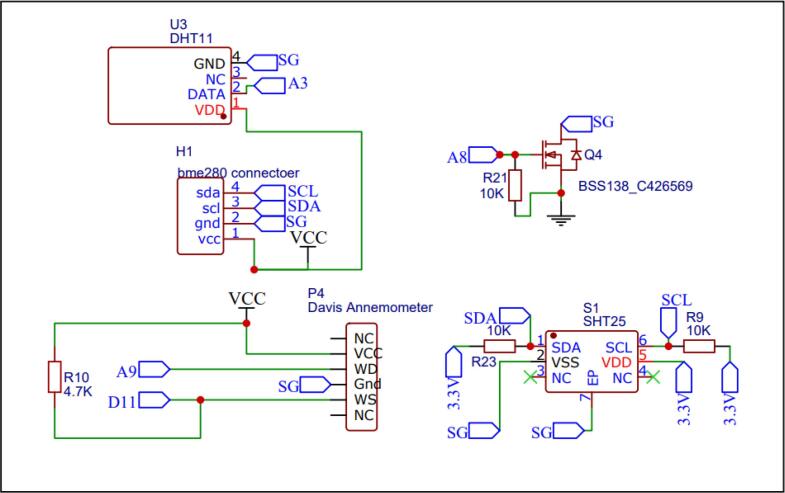
DHT, SHT, BME280 sensor schematic connections.

The AirQo sensor kit also includes a DHT11 sensor for internal relative humidity and temperature measurement. An externally placed SHT25 sensor provides external humidity and temperature data. Both sensors are connected to the main microcontroller over an i2c serial interface. An additional BME280 sensor and anemometer can also be connected through the various pin breakouts on the PCB (Fig. 5). Transistor Q4 in Fig. 5 enables the microcontroller to switch power to the sensors. Following successful data collection, data is localised and time stamped using A GPS, this data can then be stored locally on an SD card before while also being transmitted to cloud using GSM technology. Therefore, data can be stored locally on the SD-card even in areas of poor internet connectivity.

## Bill of materials

Table 2 presents a comprehensive list of components required to construct AirQo Gen 5 Rev 2 devices along with the corresponding source of materials and cost in USD.

**Table 2:** Summary of the bill of materials for the AirQo sensor kit.

**Designator**	**Component**	**Number**	**Cost per unit - (USD)**	**Total cost (USD)**	**Source of materials**	**Material type**
APINS1	Header-Male-2.54_1x5	1	0.047	0.047	lcsc.com	Metal
BATTERMINAL1	Battery connector	1	0.014	0.014	lcsc.com	Metal
BOOSTCONVERTER1	Header-Male-2.54_1x6	1	0.028	0.028	aliexpress.com	Composite
BUZZER1	ZL-YDW1207-4005PA-7.5	1	0.8	0.8	lcsc.com	Semiconductor
C1,C17,C18	22pF	3	0.025	0.075	lcsc.com	Ceramic
C2,C3,C8,C9,C14,C15,C23,C24	22uF	8	0.012	0.096	lcsc.com	Ceramic
C4	470uF	1	0.035	0.035	lcsc.com	Semiconductor
C5	470uF	1	0.1	0.1	lcsc.com	Semiconductor
C6,C7,C25	470uF	3	0.048	0.144	lcsc.com	Semiconductor
C10,C11,C12	1uF	3	0.022	0.066	lcsc.com	Ceramic
C13	33nF	1	0.022	0.022	lcsc.com	Ceramic
C19,C20,C21,C22	100nF	4	0.002	0.008	lcsc.com	Ceramic
C36	100nF	1	0.006	0.006	lcsc.com	Ceramic
CARD1	THD2528-11SD-GF	1	0.198	0.198	lcsc.com	Semiconductor
D2,D3	SR54L	2	0.148	0.296	lcsc.com	Semiconductor
D5	CD1206-S01575	1	0.039	0.039	lcsc.com	Semiconductor
D6	SK54B	1	0.135	0.135	lcsc.com	Semiconductor
DC3	DC005-2.0MM	1	0.178	0.178	lcsc.com	Metal
DPINS1	Header-Male-2.54_1x10	1	0.043	0.043	lcsc.com	Metal
GPS1 CONNECTOR	Header-Male-2.54_1x4	1	0.042	0.042	lcsc.com	Metal
GSM1 CONNECTOR	Header-Male-2.54_1x5	1	0.047	0.047	lcsc.com	Metal
H1	Header-Male-2.54_1x4	1	0.129	0.129	lcsc.com	Metal
H2	3.3 V rail	1	0.129	0.129	lcsc.com	Metal
L1,L4	4.7uH	2	0.12	0.24	lcsc.com	Semiconductor
L3	47uH	1	0.538	0.538	lcsc.com	Semiconductor
P1	Header-Male-2.54_2x3	1	0.031	0.031	lcsc.com	Metal
P2	Header-Male-2.54_1x5	1	0.035	0.035	lcsc.com	Metal
P3	Header-Male-2.54_1x2	1	0.035	0.035	lcsc.com	Metal
P4	Header-Male-2.54_1x6	1	0.071	0.071	lcsc.com	Metal
P5	Header-Male-2.54_1x2	1	0.035	0.035	lcsc.com	Metal
P6	Header-Male-2.54_1x2	1	0.035	0.035	lcsc.com	Metal
PH1	Header-Male-2.54_2x5	1	0.047	0.047	lcsc.com	Metal
POWER1	Header-Male-2.54_2x4	1	0.047	0.047	lcsc.com	Metal
Q1,Q3	IRF630NPBF	2	0.441	0.882	lcsc.com	Semiconductor
Q4,Q5,Q8,Q9,Q10	BSS138_C426569	5	0.022	0.11	lcsc.com	Semiconductor
Q6	IRF7416TRPBF	1	0.503	0.503	lcsc.com	Semiconductor
Q7	HY19P03D	1	0.35	0.35	lcsc.com	Semiconductor
R1,R2,R3,R4,R9,R21,R23,R24,R32,R33,R35,R36,R37,R38,R39,R40	10 K	16	0.001	0.016	lcsc.com	Ceramic
R5,R20,R22	73.2 K	3	0.002	0.006	lcsc.com	Ceramic
R6,R11	2 K	2	0.001	0.002	lcsc.com	Ceramic
R7,R19	10 K	2	0.002	0.004	lcsc.com	Ceramic
R8,R12,R16,R1421	1 K	4	0.001	0.004	lcsc.com	Ceramic
R10	4.7 K	1	0.004	0.004	lcsc.com	Ceramic
R13,R15,R18	20 K	3	0.182	0.546	lcsc.com	Ceramic
R17	59 K	1	0.002	0.002	lcsc.com	Ceramic
R25,R26	100 K	2	0.002	0.004	lcsc.com	Ceramic
R27,R28,R29,R30	4.7 K	4	0.001	0.004	lcsc.com	Ceramic
S1	SHT25	1	9.458	9.458	lcsc.com	Semiconductor
U1,U8	FP6298XR-G1	2	0.304	0.608	lcsc.com	Semiconductor
U2,U4	SR521 Battery Clip	2	0.149	0.298	lcsc.com	Polymer
U3	DHT11	1	1.101	1.101	lcsc.com	Semiconductor
U5	LM1117IMPX-3.3/NOPB	1	0.455	0.455	lcsc.com	Semiconductor
U6	XL4005E1	1	0.734	0.734	lcsc.com	Semiconductor
U7	DS3231MZ+	1	2.169	2.169	lcsc.com	Semiconductor
U221,U421	532,610,871 connector	2	0.209	0.418	lcsc.com	Polymer
UC1	ATMEGA 2560	1	13.5769	13.5769	lcsc.com	Semiconductor
XTAL1	16 MHz	1	0.1073	0.1073	lcsc.com	Semiconductor
PMS5003	PMS5003	2	11	22	lcsc.com	Semiconductor
DHT11	DHT11	1	1.101	1.101	lcsc.com	Semiconductor
BME5003	BME280	1	3.8	3.8	lcsc.com	Semiconductor
SHT25	SHT25	1	9.458	9.458	lcsc.com	Semiconductor
SIM800l module	SIM800l module	1	1.95	1.95	aliexpress.com	Semiconductor
NEO6 GPS module	Neo6 GPS module	1	2.58	2.58	aliexpress.com	Semiconductor
	**Total for a single unit**			**76.0422**		

## Build instructions

### PCB fabrication

Using the Gerber files, order a Printed Circuit Board (PCB) from any fabrication service provider, for example PCBWAY [28] or JLCPCB [29]. It is possible to have automated PCB assembly service to reduce build time. If this is the option, attach the bill of materials (BOM) and pick and place the file along with the order. However, if assembly is to be done manually, proceed to the next step below:

### Electronic component sourcing

Using the BOM, order the specified components from a trusted vendor for example LCSC electronics [26] or AliExpress [27]. Shopping in larger batches may yield cheaper purchase and shipping costs.

### Build process

This step is only necessary for manual assembly of the components. This step requires the following tools:

• solder rework station for surface mount components
• solder iron for through hole components
• solder flux, paste and wire.
• high precision tweezers for component placing
• PCB stencil where necessary.

**Fig. 6:**
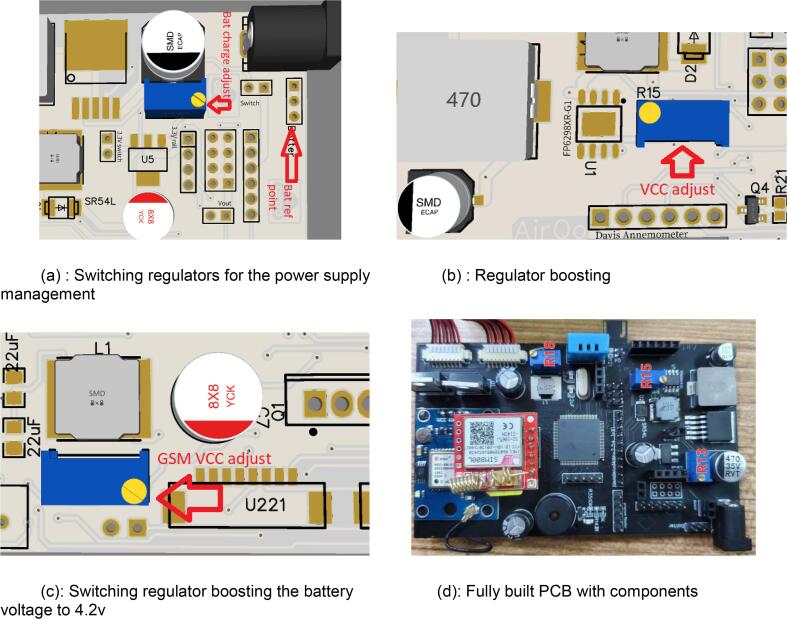
Switching regulator boosting and fully assembled PCB components.
b. 5 V is required for the ATMEGA2560 microcontroller operating at 5 V logic. This is supplied by a second switching regulator boosting the 3.7 V:4.2 V from the Li-ion battery to 5 V. This Voltage output is preconfigured in design by using appropriate resistor feedback setup, fine tuning for control (Fig. 6(b)).
c. The sim800l GSM requires 4.2 V for operation. This is provided through another switching regulator boosting the battery voltage to 4.2 V. GSM voltage output is preconfigured in design to 4.1Vby using appropriate feedback setup, fine tuning to meet network requirements (Fig. 6(c)).

The build process is as follows:

1. Apply solder flux and paste on the bare PCB pads with care.
2. Guided by the schematic and BOM, place the surface mount components on one side of the PCB and carefully apply heated air from the solder rework station.
3. Inspect the solder joints to ensure firm electrical and mechanical contact, and repeat from the bottom side.
4. solder the through hole components onto the PCB.
5. Perform voltage adjustment: the assembled PCB requires proper voltage adjustment for power domains (li-ion/li-po battery charging at 4.2 V, 5 V logic voltage for the Atmega 256 chip, 4.2 V for GSM communication component power supply). The power supply is a topology of switching regulators. Switching regulators are chosen for their high efficiency even when the drop out between the input and output is large, as compared to low dropout regulators (LDO’s) whose efficiency reduces when the input voltage is much higher than the required output voltage.
a. The voltage from the solar panel is stepped down to 4.15 V through an adjustable buck converter circuit built around the XL4005E switching regulator (Fig. 6(a)).

### Uploading and applying the firmware

Prior to deployment, firmware has to be uploaded onto the device. The process of firmware update takes the following steps:-.

1. Save the firmware as hex from Arduino
2. Connect the FTDI to the device
a. Pinout of the FTDI connection is as follows
• DTX, TX, RX, 5 V, GND
3. Using the x-loader software [30], upload the firmware onto the device. This follows the process below:-
b. Plug the USB to USART converter into the computer, and open the XLOADER tool downloaded earlier.
c. Select the device name, com port and then upload (Fig. 7a).

**Fig. 7:**
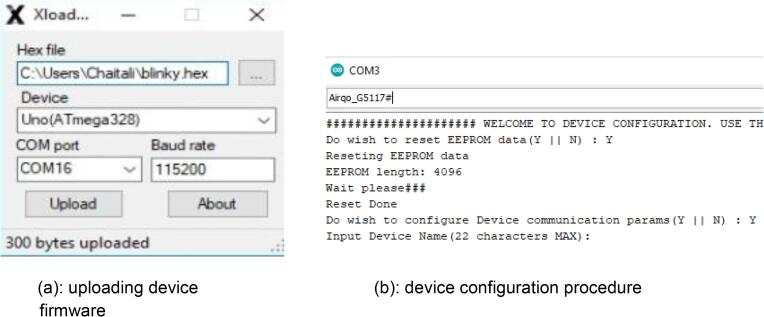
a. Uploading device firmware. b. Device configuration procedure.
4. Following a successful firmware upload. The device will require configuration which takes the following steps
d. With the FTDI connected to the device, connect pin D2 to ground: pin D2 is the first pin after the FTDI connector header pins. With D2 connected to ground, a welcome to configuration message will be printed. Type in character “y” to continue with the configuration. For every entry, “#” should be added as a delimiter (Fig. 7b).
e. You will be required to give a name to the device followed by entering the channel ID and write-API to the ThingSpeak [31] channel where data will be sent to.
f. You will also be required to fill in 0 if the device is a static, 1 for mobile and 28 as SD card CS pin number.
g. Disconnect pin D2 from ground: device details will be printed in the serial monitor followed by particulate matter concentrations.

## Operation instructions

### Deployment and installation

**Fig. 8:**
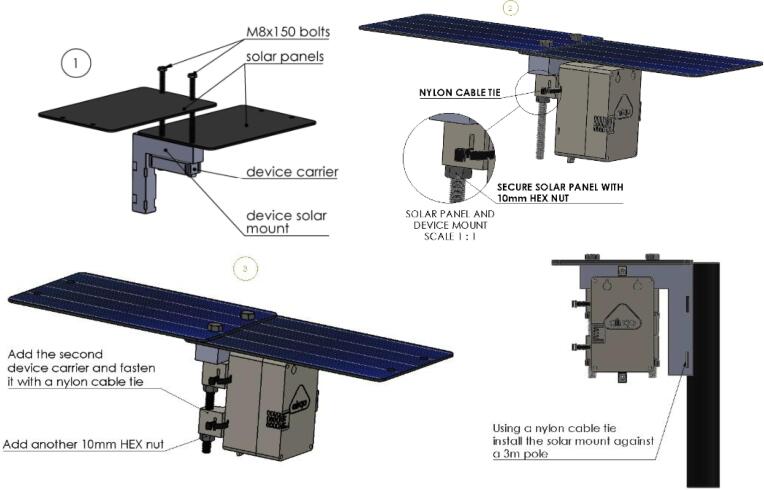
AirQo device deployment and installation procedure.

The setup and installation of any air quality sensing kit is important for meeting deployment requirements including data quality, monitoring needs, physical device security, device uptime, and personnel safety. The deployment should be preceded by siting of the location to understand the installation needs. Some sites may require a custom mounting pole to allow for monitoring without interference from the surroundings, for example, trees or buildings. We have developed a comprehensive device user guide for the proper and safer operation of the device that is available online [10]. Devices require maintenance to ensure continued reliability of the data and optimal operation of the device. The device maintenance manual is available online [11]. Fig. 8 shows the step by step procedure for installing an AirQo device in an outdoor setting.

Key considerations when deploying AirQo devices include:

• The device should be installed in free space with at least 180 degrees of air flow as surface installation causes dust to cling on the sensors which can drift and bias the readings.
• The device should not be installed close to high electric lines as they produce electromagnetic radiations which can interfere with its communication strength.
• If powering the device electrically, a 5 V, 2A charger is required.
• Upon proper installation and powering, the device will beep twice to indicate low battery and charging. Table 3 provides a summary of other beep signals made by the device and the corresponding alarms.

**Table 3:** Summary of beeping signals for troubleshooting.

**Alarms**	**Alarm Tones**	**Tone Details**	**Duration**
Power on	One beep	High	0.5 s
Transmission failure	One beep	Low	0.5 s
Low battery	Two beeps	Low High	1 s

The device records data in real time however averages after every sixty samples to send a single data sample. Depending on the battery percentage, the averaged data sample can be uploaded directly or posed up-to when five averaged data samples have been reached. On average data is transmitted to the cloud-based data store after every one and a half minutes. The monthly average data traffic per device is about 20 MB. To improve the accuracy of the measurements, the data is calibrated through co-location of a reference monitor and application of machine learning [7]. A web-based AirQo calibrate tool was developed to support calibration of the data from low-cost sensors [12]. We further describe the lab testing and field calibration of the device in the next section.

## Validation and characterization

### In-lab device testing and validation

**Fig. 9:**
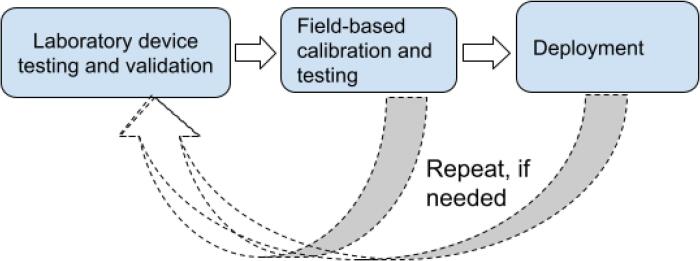
Validation and quality assurance processes of the AirQo devices.

Air quality sensors require rigorous testing and evaluation in order to ensure their accuracy and reliability. As part of the AirQo device manufacturing protocol we undertake two levels of validation and quality assurance i.e., (1) laboratory device testing and validation and (2) Field-based calibration and testing in the intended deployment settings (Fig. 9). The first step is the in-lab testing and evaluation that aims to ensure that devices collect and report data as expected. The in-lab assessment is also used to check for individual device performance as well as consistency with other devices produced in the same batch. Devices are reviewed and evaluated for any anomalies in operations that may lead to poor performance (e.g., nil data, data-out halts, delayed sensor response). At this stage any identified faults are reviewed and corrected accordingly. This may necessitate replacement of electronic components and modifications to the firmware. Devices are also reviewed and assessed for proper and quality data output for GSM data, GPS data, dual PM Sensors data, temperature humidity sensors data, power and voltage readings. We undertake intra-device assessment to confirm that dual sensors are consistent in measurements. Further, we compare each device with other devices in the same batch to ensure consistency. Devices that pass are moved to the next stage of the evaluation. A selection of the devices that pass the laboratory testing and validation is moved to the field-based calibration and testing from where a calibration function is developed and applied to all devices in the same batch. We describe the field-based calibration and testing process in the next subsection.

As an example, experiment setup for in-lab validation and analysis, we consider six AirQo devices that are part of the same manufacturing batch. The six devices are identified as Airqo_G5111, Airqo_G5115, Airqo_G5127, Airqo_G5130, Airqo_G5131, and Airqo_G5133. The preceding pm2_5 or pm2_10 indicates the parameter being analysed, that is, PM_2.5_ and PM_10_, respectively. Table 4 shows an example of correlation between the devices.

**Table 4:** Example analysis of in-lab validation and testing of six devices for PM_2.5._

**device**	**pm2_5(Airqo_G5111)**	**pm2_5(Airqo_G5115)**	**pm2_5(Airqo_G5127)**	**pm2_5(Airqo_G5130)**	**pm2_5(Airqo_G5131)**	**pm2_5(Airqo_G5133)**
**pm2_5(Airqo_G5111)**	1.0	0.9862	0.9962	0.9950	0.9924	0.7923
**pm2_5(Airqo_G5115)**	0.9862	1.0	0.9863	0.9928	0.9938	0.6562
**pm2_5(Airqo_G5127)**	0.9962	0.9863	1.0	0.9954	0.9891	0.6659
**pm2_5(Airqo_G5130)**	0.9950	0.9928	0.9954	1.0	0.9973	0.6624
**pm2_5(Airqo_G5131)**	0.9924	0.9938	0.9891	0.9973	1.0	0.6468
**pm2_5(Airqo_G5133)**	0.7923	0.6562	0.6659	0.6624	0.6468	1.0

**Fig. 10:**
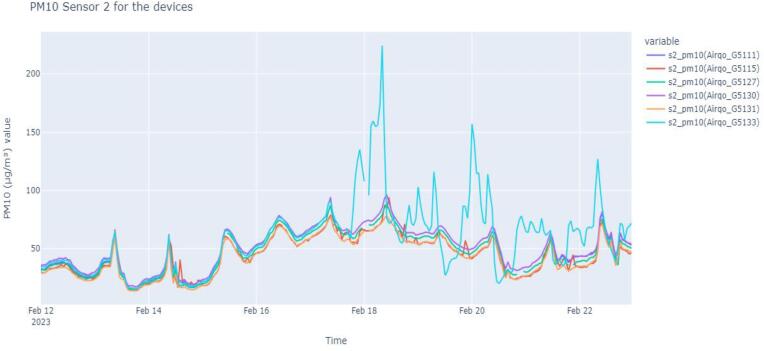
Example analysis of in-lab validation and testing of six devices for PM_10._

Fig. 10 shows the analysis for PM_10_ for the six devices. From the analysis, it can be observed that the device Airqo_G5133 needs further review before proceeding to the next step.

### Field-based calibration and testing

The performance of low-cost air quality sensors may be affected by external operating conditions such as temperature and relative humidity [7]. As such it is important to embedded quality assurance processes in the workflow and data pipeline of low-cost air quality sensors. Embedded calibration methods and quality assurance is a major design consideration for the AirQo devices [5].

**Fig. 11:**
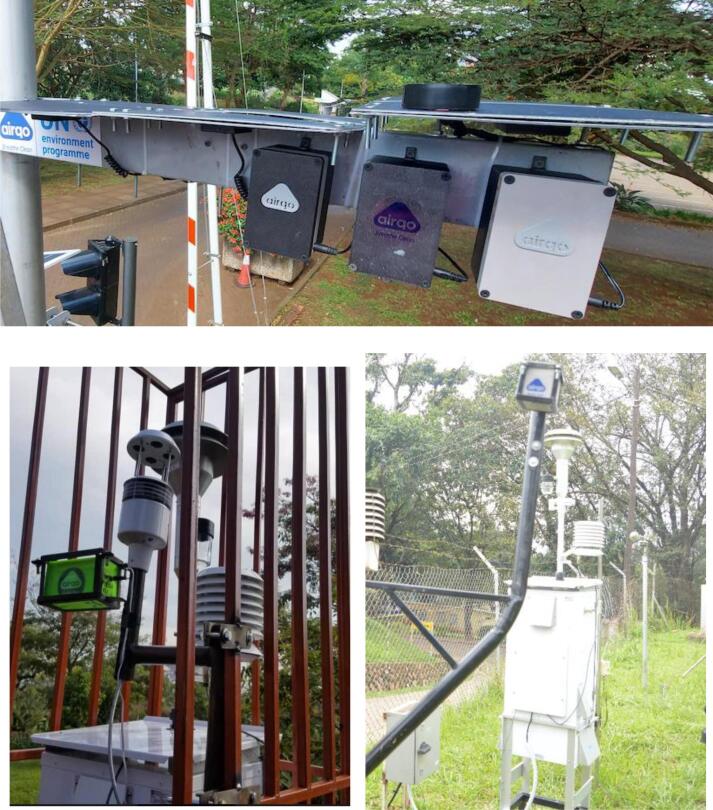
Field co-location of the AirQo devices with the reference grade monitor.

To assess and validate the performance of the AirQo devices, we co-locate them with the reference grade monitors in the field. Reference grade monitors are designated by regulatory authorities and agencies as having high precision and accuracy and thus can be used for regulatory purposes. Countries typically define national air quality regulations and standards that define acceptable or health levels for various pollutants. The WHO provides guidelines on the acceptable and target levels for various pollutants aimed at protecting public health from the dangers of air pollution [13]. For some countries these regulations are still under development or not yet developed. The national air quality regulations and standards also define the monitoring standards and equipment. Some of the commonly used reference grade monitors are beta attenuation monitor (BAM) and tapered element oscillating microbalance (TEOM) [5], [14]. We selected the beta attenuation monitor (BAM1022) as the reference monitor because it is designated by the regulatory agencies such as the U.S. Environmental Protection Agency (EPA) as a monitoring equipment that meets the requirements of the federal equivalent method and we have access to the equipment [15]. Fig. 11 shows an example co-location site including the AirQo devices and the reference grade monitor in an outdoor setting.

### Performance in comparison with the reference grade monitor

**Fig. 12:**
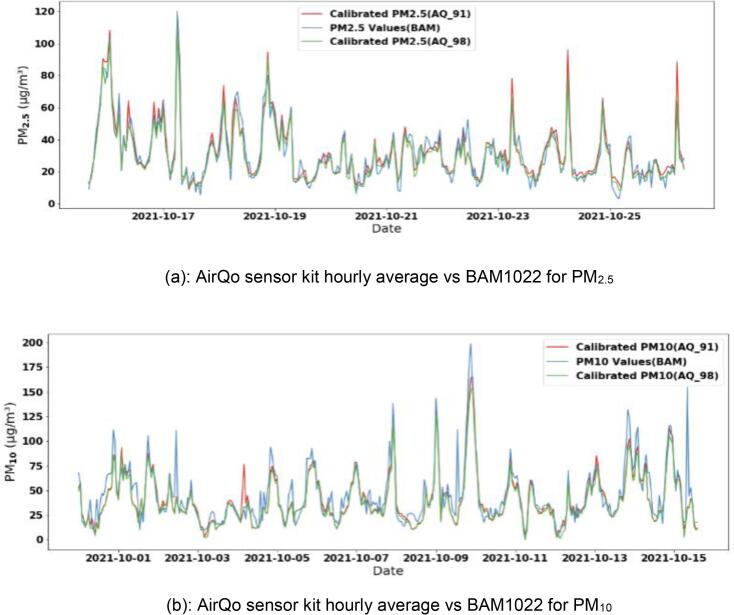
Comparison of AirQo devices with the reference grade monitor BAM1022 [8].

AirQo devices track strongly with the reference grade beta attenuation monitor (BAM1022) as shown in (Fig. 12). The AirQo devices have been co-located with the BAM1022 devices for PM_2.5_ and PM_10_. The correlation between AirQo devices and BAM1022 before calibration is 0.9. We use the datasets from the co-location of the low-cost sensors and the reference grade monitors to develop a machine learning-based calibration model.

**Fig. 13:**
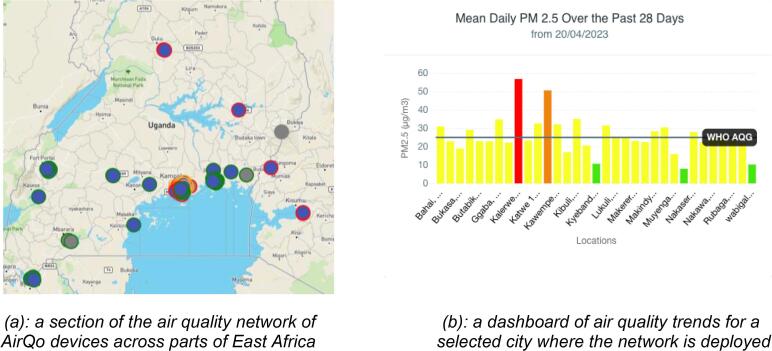
Example use case of the AirQo sensor kit in a low-resource setting.

For the calibration model and validation experiments presented in the above analysis, we considered a co-location dataset from over a period of seven months. The dataset consists of an average hourly value from the AirQo device and a corresponding average value from the BAM1022. We randomly selected 80 % of the dataset as a training set and the remaining 20 % as the validation set. The accuracy of AirQo devices is further enhanced by machine learning-based calibrations methods. Following the performance evaluation of different machine learning algorithms, we selected random forest and lasso regression algorithms as the most suitable. In addition to the average particulate matter hourly values, we considered meteorological factors (temperature and relative humidity) as input variables to the model. Temperature and relative humidity directly impact on the characteristics of the particulate matter pollutants and their operating principle of that is based on the laser scattering technique for the measurement of particle size [5]. The machine learning calibration model is described in detail in [8]. After calibration, the correlation between AirQo devices and BAM1022 improves to 0.96 and the RMSE is 7.22 μ*g*/*m*^3^ for PM_2.5._ and 7.9 μ*g*/*m*^3^ for PM_10._ Fig. 13(a) and (b) show the BAM1022 comparison of the reference monitor and the AirQo sensors for PM_2.5._ and PM_10_.

### Relevant use case

The AirQo sensor system has been deployed widely in cities in Africa. Fig. 13(a) shows a snapshot of a section of East Africa where devices are deployed. In many of these cities there is no continuous air quality monitoring and as such the AirQo network has initiated the ground work in air quality monitoring, awareness, education and policy. The AirQo system includes a digital dashboard for city authorities and a mobile app for citizens, providing data analytics on the state of air quality in areas of interest. Fig. 13(b) shows a dashboard for a selected city.

**Fig. 14:**
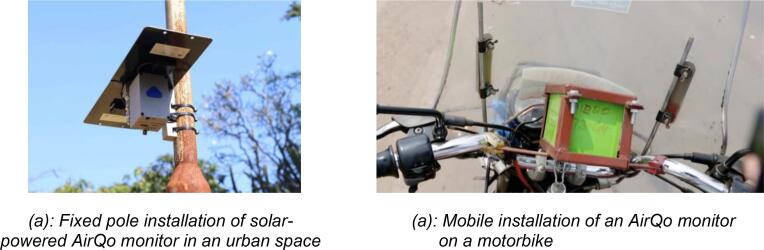
Fixed and mobile installation examples of the AirQo sensor kit east African cities (Kisumu, Kenya and Kampala, Uganda respectively).

The datasets from the AirQo network has informed several scientific studies including characterization of ambient air quality in several urban towns in Uganda [16], estimation of PM2.5 using satellite datasets [17], understanding the impacts Covid-related lockdowns on air quality [18], enabling land use regression models [19], Uganda national state of environment report [20], machine learning data challenges [21], [22], studying social vulnerability for air pollution [23], and studying the interplay between air pollution and mobility [24], among others. Fig. 14(a) and (b) show the deployment of AirQo devices on fixed and mobile locations.

## Discussion and conclusion

In this paper, we have described the construction of the AirQo sensor kit, a lower cost air quality sensing kit specially designed and inspired by the operating conditions from African cities. The focus on African cities is motivated by the lack of sufficient air quality and environmental monitoring datasets at a higher resolution and longer time. Informed by the practical experiences and understanding of the operating contexts, the paper provides step by step instructions on the design, construction, calibration and deployment of the sensing device. The AirQo sensor has been deployed in the field and validated against the reference grade monitor, and shows agreement and improved performance with the machine learning-based calibration methods.

Beyond the hardware development, the paper provides characterisation of the use cases and deployment of a large-scale air quality sensing system in several African cities and countries. Particulate matter is considered a major indicator of quality ambient air quality. This is because of the available evidence on its implications on human health. As such the AirQo devices are optimised to help developing countries quantify particulate and collect evidence that can inspire action. The AirQo device is premised on modular design principles allowing for addition of other parameters. The future plans include expansion of the AirQo devices to include other criteria gaseous pollutants such as Nitrogen dioxide, sulphur dioxide, and ozone. In the future iterations, the AirQo sensing kit will be extended to include other major pollutants such as NOx, SOx, and Ozone.

The current AirQo sensor leverages a cloud-based calibration model. While this approach serves for the purpose of calibrating data from a large-scale network, there is also a need to consider purely offline on device calibration models, for personalised monitoring. The AirQo sensor kit has been validated and optimised for outdoor ambient air quality monitoring. However, low- and middle-income countries also experience indoor pollution problems. In future iterations, the AirQo sensor kit needs to be further refined to consider personalised and indoor air quality monitoring. Therefore, future iterations of the AirQo sensor kit model are expected to meet the growing needs of air quality monitoring in low-resource settings. It is expected that the hardware and software infrastructure presented in this paper will inspire other researchers to build and customise need-specific devices for contexts where other pollutants may be considered a higher priority.

## Ethics statements

This work does not involve human or animal subjects.

## CRediT authorship contribution statement

**Engineer Bainomugisha:** Conceptualization, Methodology, Funding acquisition, Investigation, Supervision, Writing – original draft, Writing – review & editing. **Joel Ssematimba:** Validation, Conceptualization, Software, Writing – review & editing. **Deogratius Okedi:** Software, Data curation, Writing – review & editing. **Marvin Banda:** Data curation, Writing – review & editing. **George William Settala:** Data curation, Writing – review & editing. **Gideon Lubisia:** Methodology, Writing – review & editing.

## Declaration of Competing Interest

The authors declare that they have no known competing financial interests or personal relationships that could have appeared to influence the work reported in this paper.
